# Mexico as a hotspot for plant virus evolution: eco-evolutionary regimes shaping viral emergence

**DOI:** 10.3389/fpls.2026.1800439

**Published:** 2026-04-07

**Authors:** Flor C. Alcántar-Aguirre, Erika J. Zamora-Macorra, Ubilfrido V. Gutiérrez, Jimena Carrillo-Tripp, Alfredo Diaz-Lara, Katia Aviña-Padilla

**Affiliations:** 1 Department of Developmental Neurobiology and Neurophysiology, Institute of Neurobiology, Universidad Nacional Autónoma de Mexico, Queretaro, Mexico; 2 Department of Agricultural Preparatory School, Universidad Autónoma Chapingo, Texcoco, Estado de México, Mexico; 3 Department of Parasitology, Universidad Autonoma Agraria Antonio Narro, Saltillo, Mexico; 4 Department of Microbiology, Centro de Investigación Científica y de Educación Superior de Ensenada (CICESE), Ensenada, Baja California, Mexico; 5 School of Engineering and Sciences, Tecnologico de Monterrey, Queretaro Campus, Santiago de Queretaro, Queretaro, Mexico; 6 Department of Cell Biology, Centro de Investigación y de Estudios Avanzados del Instituto Politecnico Nacional, Mexico City, Mexico

**Keywords:** agricultural epidemics, genomic variation, molecular epidemiology, plant virus evolution, viroids, virus emergence

## Abstract

Plant virus and viroid emergence in Mexico reflects the operation of distinct eco-evolutionary regimes rather than isolated epidemiological events. Mexico’s pronounced agroecological heterogeneity, together with its status as a center of crop domestication and diversification, has generated long-term arenas for virus–host–vector coevolution, repeated viral introductions, and rapid adaptive diversification. In this perspective, we synthesize historical, epidemiological, and molecular evidence and organize it into three dominant eco-evolutionary regimes: endemic persistence shaped by long-term coevolution, evolutionary entrenchment following viral invasion, and rapid emergence driven by agricultural intensification and system connectivity. Case studies spanning annual and perennial crops illustrate how vector ecology, host genetic diversity, and production practices interact to shape viral evolutionary trajectories and epidemiological outcomes. Collectively, these regimes explain recurring patterns of plant virus and viroid emergence across Mexican agroecosystems and reveal shared evolutionary mechanisms underlying both long-term endemic stability and episodic epidemic outbreaks. We argue that recognizing and integrating these eco-evolutionary regimes provides a predictive framework for plant virus surveillance and embeds evolutionary principles into disease prevention and management strategies, not only in Mexico but in agroecological systems worldwide where similar structural and connectivity dynamics shape viral diversification.

## 1 Introduction: Mexico as an evolutionary hotspot for plant viruses

Mexico has recurrently experienced the disruptive impacts of plant virus and viroid epidemics, which continue to threaten crop productivity, food security, and the economic stability of key agricultural regions. The country’s exceptional agroecological heterogeneity, spanning tropical lowlands, temperate zones, and semi-arid highlands, combined with a mosaic of traditional, semi-intensive, and industrial production systems, creates an ecological context particularly conducive to viral persistence, diversification, and emergence. Within this landscape, virus and viroid-associated diseases increasingly affect both staple and high-value crops, underscoring the need for an integrated evolutionary perspective to understand plant viral dynamics in Mexico (García-Estrada et al., 2022). This country constitutes a unique natural laboratory for the study of plant virus evolution. As a recognized center of domestication for crops such as maize (*Zea mays*), chili pepper (*Capsicum annuum*), and squash (*Cucurbita* spp.), the country harbors extensive genetic diversity within cultivated plants and their wild relatives.

**Figure 1:**
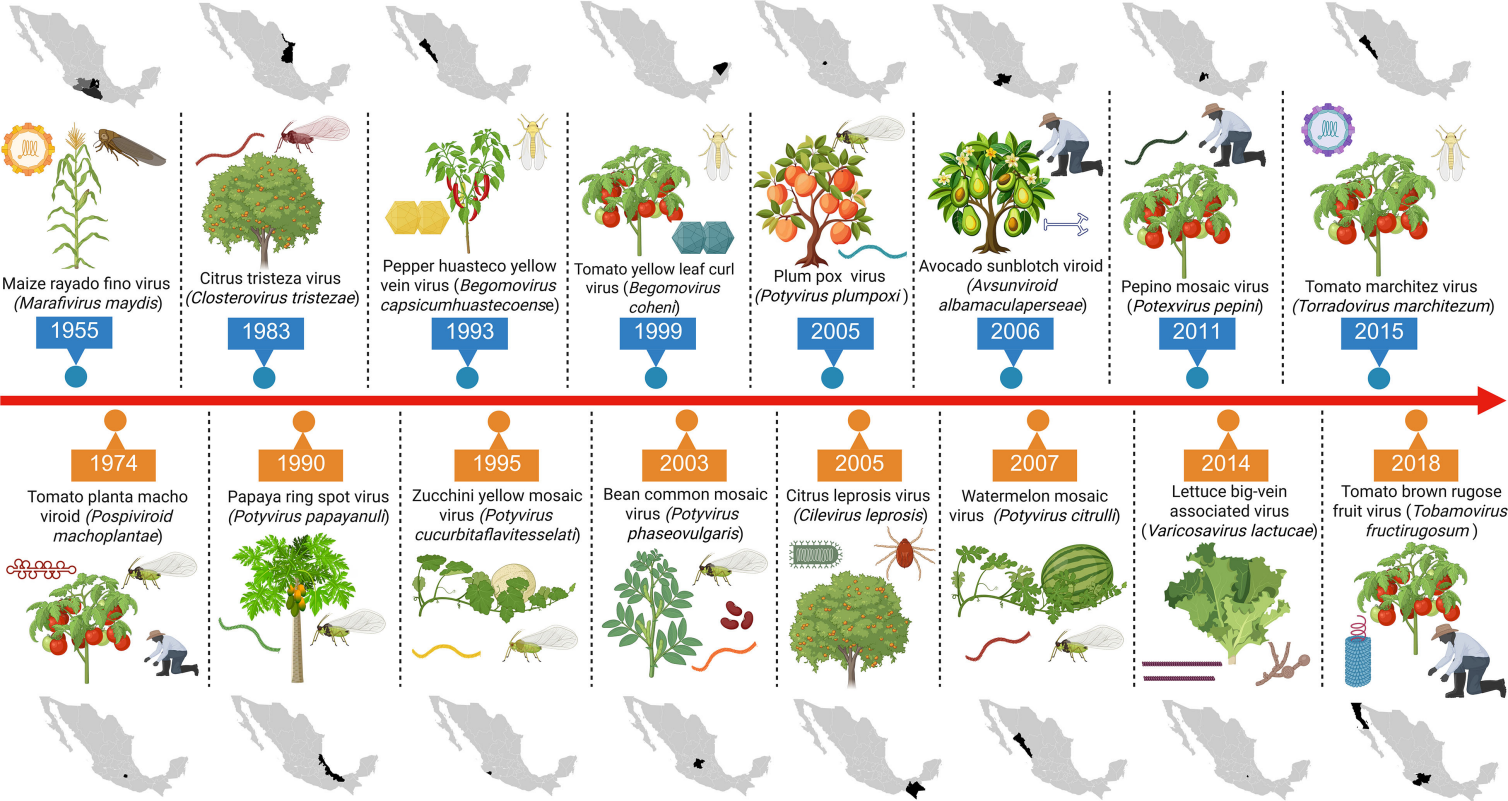
Timeline of the emergence and introduction of major plant viruses and viroids affecting crops in Mexico.The timeline depicts the chronological detection of representative plant viruses and viroids reported in Mexico between 1955 and 2018.Created at https://BioRender.com.

This diversity, together with a wide array of insect vectors, generates multiple ecological interfaces in which viruses can persist, adapt, recombine, and undergo host-range shifts. These processes are further intensified by contemporary drivers including agricultural intensification, climate variability, and the sustained movement of plant material across regional and international boundaries, all of which enhance opportunities for viral introduction, recombination, and epidemic establishment (Morris and Moury, 2019; Butković and González, 2022). Historical records indicate that plant virus epidemics have recurrently shaped Mexican agriculture throughout the twentieth and twenty-first centuries, revealing persistent structural vulnerabilities associated with monoculture expansion, vector-mediated transmission, and the globalization of seed and plant trade (**Figure 1**).

Although plant viruses and subviral agents have likely circulated in Mexican agroecosystems since the early stages of agriculture, the systematic documentation of large-scale viral and viroid epidemics is relatively recent. During the first half of the twentieth century, phytosanitary efforts in Mexico were almost exclusively focused on fungal and bacterial diseases. A representative example is the implementation, during the 1930s, of chemical control campaigns using Bordeaux mixture against *“Chamusco del plátano”* in banana plantations in southeastern Mexico, particularly in the state of Tabasco (Stern, 1938). These actions coincided with the institutionalization of plant pathology and phytosanitary science, marked by the establishment of academic and governmental bodies such as the National School of Agriculture at Chapingo and early national plant health authorities (Cañedo, 1988; Rodríguez Vallejo, 2000; Camacho Morales et al., 2025).Throughout the mid-twentieth century, Mexican phytopathology underwent a phase of scientific consolidation driven by crop improvement programs, disease surveillance initiatives, and the strengthening of national institutions devoted to agricultural production and plant health. Nevertheless, plant viral and viroid diseases were largely underestimated, addressed primarily in descriptive or localized studies and lacking a national epidemiological framework (Galindo Mendoza and Contreras Servín, 2017; Servín, 2022). As a result, epidemic-scale outbreaks of plant viruses and viroids were not clearly recognized until the late twentieth century.

The first well-documented viral outbreak in Mexican agriculture corresponds to the detection of Citrus tristeza virus (CTV; *Closterovirus tristezae*), reported in 1983 in the state of Tamaulipas and subsequently in Veracruz in 1986, where early incursions were eradicated through phytosanitary measures (Iracheta-Cárdenas et al., 2010). During the 1990s, the plant virus landscape in Mexico changed markedly. Papaya ringspot virus (PRSV; *Potyvirus*) became widely distributed across papaya-producing regions and emerged as a major constraint to crop productivity (Noa-Carrazana et al., 2006). In parallel, the first detection of Zucchini yellow mosaic virus (ZYMV; *Potyvirus*) was reported in melon crops in Colima in 1994 (Orozco-Santos et al., 1994). A major turning point occurred in the late 1990s with the emergence and rapid spread of whitefly-transmitted begomoviruses. Among them, Pepper huasteco yellow vein virus (PHYVV; *Begomovirus capsicumhuastecoense*) became one of the most epidemiologically relevant pathogens in pepper production systems. In Sinaloa, field surveys of symptomatic *Capsicum annuum* plants revealed PHYVV infection in 74.4% of the samples, highlighting its dominant role in disease outbreaks (Hernández-Espinal et al., 2018). PHYVV is associated with severe yield and quality losses, shows broad distribution throughout Mexico and Central America, and is transmitted by *Bemisia tabaci* (Retes-Manjarrez et al., 2018). During the 2000s, surveys conducted in the Mesoamerican center of domestication documented the presence of multiple viruses infecting common bean (*Phaseolus vulgaris*), including Bean common mosaic virus (*BCMV; genus Potyvirus*), Bean common mosaic necrosis virus *(BCMNV; genus Potyvirus*), Bean golden yellow mosaic virus *(BGYMV; Begomovirus phaseoli)*, and Cowpea mild mottle virus *(CPMMV; genus Carlavirus)*, particularly in the states of Guanajuato, Jalisco, and Nayarit (Chiquito-Almanza et al., 2021). In the last decade, the diversity of emerging plant viruses and viroids in Mexico has continued to expand, with reports of Pepino mosaic virus (*PepMV; genus Potexvirus*) in greenhouse tomatoes (Verbeek et al., 2014), Lettuce big-vein associated virus *(LBVaV; genus Varicosavirus)* (2014), Tomato marchitez virus (2015) (Verbeek et al., 2008), and Tomato brown rugose fruit virus (*ToBRFV; genus Tobamovirus*) (2019).

Beyond plant viruses, subviral agents such as viroids have played a significant and historically underappreciated role in Mexican agroecosystems. Viroids are small, single-stranded circular RNA molecules capable of infecting a wide range of crop species, frequently inducing severe developmental and physiological alterations despite their minimal genomic complexity (Aviña-Padilla et al., 2022). Mexico constitutes a geographical landscape of exceptional viroid diversity and is recognized as a center of origin for multiple viroid species of epidemiological and economic relevance, reflecting long-term evolutionary interactions between viroids and native or long-cultivated hosts (Aviña-Padilla et al., 2022). Among the *Pospiviroidae*, Tomato planta macho viroid (TPMVd; genus *Pospiviroid*, family *Pospiviroidae)* represents one of the earliest viroids characterized in the country. The disease known as “planta macho” was first described in Mexico by Galindo and collaborators in the early 1980s, who identified TPMVd as the causal agent and documented its association with severe symptoms including stunting, epinasty, chlorosis, leaf malformation, and reduced fruit set and yield in tomato crops (Galindo and Rodríguez, 1978). Subsequent molecular analyses demonstrated that *Mexican papita viroid* (MPVd), identified in wild *Solanum* species, is genetically indistinguishable from TPMVd, supporting their classification as variants of a single species and underscoring their prominence in solanaceous cropping systems in Mexico (Aviña-Padilla et al., 2022). In addition to TPMVd, Avocado sunblotch viroid (ASBVd; genus *Avsunviroid*, family *Avsunviroidae*), a member of the *Avsunviroidae*, constitutes another viroid of major agricultural significance endemic to Mexican agroecosystems. ASBVd infects avocado (*Persea americana*) and is associated with sunken longitudinal streaks on fruit, color break, shoot striping, and substantial yield reductions, frequently exceeding 30%, even in trees that remain asymptomatic for extended periods (Aviña-Padilla et al., 2022). The endemic occurrence of TPMVd/MPVd in tomato and ASBVd in avocado highlights the central role of Mexico in the evolutionary history of viroids and emphasizes the need to explicitly incorporate subviral agents into plant disease surveillance, epidemiology, and management strategies alongside plant viruses.

Concurrently, advances in high-throughput sequencing and viral metagenomics have fundamentally reshaped the study of plant virus and viroid diversity in Mexico, revealing complex population structures, frequent mixed infections, and extensive circulation across crops, weeds, insect vectors, and environmental reservoirs (Massart et al., 2017; Pacheco-Dorantes et al., 2025). Collectively, this historical and epidemiological evidence indicates that plant virus and viroid epidemics in Mexico are not isolated events but rather the outcome of long-term eco-evolutionary processes, shaped by agroecological heterogeneity, agricultural intensification, and the movement of infected propagative material. This framework provides the necessary context for interpreting the patterns of viral and viroid emergence and persistence examined in this study.

## 2 Endemic viral and subviral agents long-term coevolution

Maize rayado fino virus (MRFV; *Marafivirus maydis*) is a paradigmatic example of endemic plant virus evolution in Mesoamerica, where long-term persistence is tightly linked to the biocultural history of maize cultivation molecular, epidemiological, and phylogenetic evidence supports a Mesoamerican; Mexico and/or Guatemala origin of MRFV, followed by regional dispersal within the Americas, rather than recent global introduction events (Hammond et al., 1997; Chicas et al., 2007). MRFV shows a narrow host range restricted to *Zea* species and is transmitted by the maize-specialist leafhopper *Dalbulus maidis*, reflecting a long-standing coevolutionary association among virus, host, and vector (Hammond and Ramírez, 2001; Gray and Banerjee, 1999). Genomic studies reveal low nucleotide diversity and strong purifying selection, consistent with evolutionary stability and spatially structured populations shaped by local adaptation rather than recurrent invasion (Hammond and Ramírez, 2001; García-Arenal et al., 2001) Chicas et al., 2007). In Mexico, contrasting agroecosystems, from traditional milpa systems to intensified monocultures, modulate MRFV epidemiology without altering its fundamentally endemic character, illustrating how agricultural context shapes virus persistence and expression (Roossinck, 2011).

In the same context, Avocado sunblotch viroid (ASBVd; *genus Avsunviroid, family Avsunviroidae*) exemplifies extreme latent persistence and long-term evolutionary stability in perennial crops. Its epidemiological success relies on vegetative propagation, prolonged asymptomatic infections, and host longevity, enabling cryptic accumulation and dissemination over decades (Flores et al., 2005). First reported in Mexico in 2006–2007 in Michoacán, many infected avocado trees remain symptomless while acting as persistent inoculum sources, making ASBVd a paradigmatic case of latent emergence in a globally strategic crop (De La Torre-A et al., 2009). ASBVd shows low genetic diversity, consistent with strong structural constraints imposed by hammerhead ribozyme-based replication, yet stable variants indicate long-term local diversification and possible endemism linked to the Mesoamerican origin of avocado (Saucedo Carabez et al., 2019).This evolutionary pattern parallels that of tomato planta macho viroid (TPMVd; genus *Pospiviroid*, family *Pospiviroidae*) and its wild relative Mexican papita viroid (MPVd), which persist asymptomatically in native hosts and support a model of viroid emergence from endemic reservoirs followed by anthropogenic dissemination (Galindo et al., 1982).; (Martinez-Soriano et al., 1996) (Verhoeven et al., 2011) Diener, 2001). Together, ASBVd and TPMVd/MPVd highlight Mexico as a center of origin and diversification for agriculturally relevant viroids, exemplifying an eco-evolutionary regime dominated by persistence, latency, and slow diversification rather than acute epidemic spread.

Another case is Pepper huasteco yellow vein virus (PHYVV; *Begomovirus capsicumhuastecoense*), a paradigmatic example of endemic begomovirus evolution in Mexico shaped by long-term host association, persistent whitefly-mediated transmission, and stable agroecological connectivity. First characterized in pepper crops from the Huasteca region, PHYVV displays high genetic similarity across Mexican regions, supporting long-term endemic persistence with gradual regional diversification rather than recent introduction (Torres-Pacheco et al., 1993) (Hernández-Espinal et al., 2018). A defining feature of PHYVV is its recurrent participation in mixed infections with Pepper golden mosaic virus (PepGMV), which generate synergistic interactions that intensify disease severity, increase viral accumulation, and suppress host recovery (Rentería-Canett et al., 2011; Rodríguez-Gandarilla et al., 2020). These mixed infections facilitate recombination and genetic exchange, reinforcing their role as stable drivers of begomovirus diversification within endemic Mexican pepper agroecosystems (Méndez-Lozano et al., 2003). In this sense, PHYVV does not merely persist as an isolated lineage but participates in a structured, coevolving viral community, where recombination and synergy operate within a relatively stable epidemiological framework. As in the case of MRFV, long-term ecological embedding rather than episodic invasion defines its evolutionary trajectory.

## 3 Introduced viruses: invasion, adaptation, and evolutionary entrenchment

In contrast to endemic systems, Tomato yellow leaf curl virus (TYLCV; *Begomovirus coheni*) represents a paradigmatic example of a globally invasive begomovirus that has become firmly established in Mexico. Originating in the Middle East–Mediterranean region, TYLCV diversified in association with tomato cultivation and *Bemisia tabaci* populations prior to its global dissemination (Moriones and Navas-Castillo, 2010; Lefeuvre et al., 2010). Its epidemiological success in Mexico has been strongly facilitated by the expansion of invasive *B. tabaci* cryptic species *MEAM1* and *MED*, characterized by high dispersal capacity and exceptional transmission efficiency (De Barro et al., 2011). Under conditions of continuous tomato production, high plant turnover, and monoculture practices, TYLCV rapidly established across multiple agroecological regions.

Importantly, following its introduction, frequent mixed infections with native New World begomoviruses promoted recombination and genetic exchange, accelerating adaptive integration into local viral communities. Thus, TYLCV does not represent merely an invasive episode but rather a case of invasion followed by evolutionary stabilization, where rapid initial expansion transitions into long-term ecological embedding. In the same context of invasion-driven establishment, Citrus tristeza virus (CTV; *Closterovirus tristezae*) exemplifies evolutionary entrenchment following introduction into perennial cropping systems. Early surveys in Nuevo León (1995–1998) detected CTV in 0.25% of 18,950 citrus trees, with grove-level incidence of 4.34%, 0.02%, and 0.70% (Silva-Vara et al., 2001). Although 35 of 47 infected trees reacted to the decline-specific MCA13 antibody, none displayed visible symptoms, revealing the coexistence of mild and decline-inducing isolates within the same production landscape. Subsequent surveys documented CTV in up to 20 Mexican states, frequently without overt symptom expression even on susceptible sour orange rootstocks (Iracheta-Cárdenas et al., 2010; Herrera-Isidrón et al., 2009). Molecular analyses identified two major genetic groups, mild and severe isolates, whose divergence correlated with pathogenicity, while disease expression was strongly modulated by host genotype, rootstock–scion combinations, and orchard management practices (Rocha-Peña et al., 1995; Moreno et al., 2008; Herrera-Isidrón et al., 2009).

Together, TYLCV and CTV illustrate a shared trajectory of introduction followed by long-term integration, yet under distinct ecological architectures. Whereas TYLCV established within annual, high-turnover cropping systems characterized by rapid epidemiological cycles, CTV became embedded within perennial orchard ecosystems where host longevity, clonal propagation, and management regimes favor gradual coexistence and cryptic circulation. In contrast to rapidly spreading annual-crop epidemics, CTV dynamics reflect long-term persistence, genotype-by-environment modulation of virulence, and selection for compatibility rather than acute epidemic explosiveness.

## 4 Emerging viruses in annual crops: rapid evolution under agricultural intensification

If perennial systems favor gradual integration and long-term coexistence, annual cropping systems represent highly dynamic evolutionary arenas for plant viruses. Short host life cycles, high planting density, genetic uniformity, and continuous crop turnover impose recurrent transmission bottlenecks and strong selection for transmission efficiency, host adaptation, and resistance breaking (Hanssen and Thomma, 2010; Elena et al., 2014; Tatineni and Hein, 2023; Vásquez Gutiérrez et al., 2025a). In Mexico, the expansion of intensive and semi-intensive agriculture has increased landscape connectivity through seedlings, seed lots, and synchronized production cycles, amplifying viral introductions and accelerating epidemic growth (Elena et al., 2014; García-Estrada et al., 2022; Vásquez-Gutiérrez et al., 2025a).

ToBRFV exemplifies recent emergence under these conditions (Salem et al., 2016). First detected in Mexico in 2018–2019 in Baja California and Michoacán, this virus rapidly spread nationwide, reflecting high environmental stability, efficient mechanical and seed transmission, and repeated epidemiological bottlenecks (Salem et al., 2016; Camacho-Beltrán et al., 2019; Cambrón-Crisantos et al., 2019; van de Vossenberg et al., 2020; Fidan et al., 2024). Despite the low overall genetic diversity, genomic analyses revealed host-associated single-nucleotide variants concentrated in the replicase methyltransferase domain, with distinct profiles across *Solanum lycopersicum*, *Capsicum annuum*, *Solanum nigrum*, and *Citrullus lanatus*, suggesting host-specific selective pressures and multiple introduction events (Zamora-Macorra et al., 2025b; García-Andrés et al., 2006).

Cucurbit systems further illustrate accelerated evolution in annual crops. In northwestern Mexico, Zucchini yellow mosaic virus (ZYMV) dominates cucurbit viromes, with frequent coinfections involving PRSV-W and WMV documented since its first report in melon in 1994 (Orozco-Santos, 1994; Morales and Jones, 2004). WMV diversification is strongly shaped by recombination, while ZYMV exhibits rapid mutation-driven evolution that undermines resistance durability (Desbiez and Lecoq, 2004; Simmons et al., 2008). The recent confirmation of Cucurbit aphid-borne yellows virus (CAByV) via HTS underscores the expanding viral complexity of cucurbit systems in Mexico (Rabadán and Gómez, 2023; Vidal et al., 2023).

Soilborne emergence is exemplified by Lettuce big-vein associated virus (LBVaV), first reported in Mexico City with field losses reaching 93%, consistent with early-stage establishment in intensive horticulture (Ochoa-Martínez et al., 2014). Transmission by *Olpidium* spp. enables long-term soil persistence and repeated bottlenecks, making eradication difficult once fields are infested (Sasaya et al., 2008; Mavrič Pleško et al., 2009).

Protected horticulture represents an extreme intensification regime. In Mexican greenhouses, Tomato marchitez virus (ToMarV) caused up to 100% seedling mortality and yield losses approaching 60%, reflecting rapid amplification under dense planting and mechanical handling (Verbeek et al., 2008). Similarly, Pepino mosaic virus (PepMV) has become entrenched in protected tomato systems since its report in Mexico in 2011, with multiple genotypes co-circulating and yield losses reaching 40% in severe outbreaks (Ling and Zhang, 2011; Verbeek et al., 2014; Hanssen and Thomma, 2010).

Bean common mosaic virus (BCMV) illustrates resistance-driven emergence in annual legumes. Seed transmission and aphid-mediated spread, combined with recurrent breakdown of the classical I resistance gene, have generated resistance-breaking strains associated with yield losses of up to 85% when infection occurs early in crop development (Mandour et al., 2013). Surveys across major bean-producing regions in Mexico confirm widespread infection in both cultivated and wild *Phaseolus* species, underscoring the role of seed trade, host selection, and landscape connectivity as drivers of ongoing diversification (Chiquito-Almanza et al., 2021). The interplay between resistance deployment and viral counteradaptation exemplifies a feedback loop in which breeding strategies inadvertently create strong selective pressures favoring virulent variants, thereby accelerating evolutionary turnover (Elena et al., 2014).

Together, these systems demonstrate that annual cropping landscapes in Mexico function as evolutionary accelerators. Rapid mutation, recombination, recurrent transmission bottlenecks, and high epidemiological connectivity collapse evolutionary and epidemiological timescales, producing dynamic viral populations capable of swift adaptation (Dawson et al., 2015). In such contexts, resistance durability, seed certification, and vector management must be designed within explicitly evolutionary frameworks rather than reactive outbreak-based models.

## 5 Discussion

The evidence synthesized in this communication demonstrates that plant virus emergence in Mexico cannot be adequately explained by linear diffusion models or single-cause frameworks. Instead, emergence and diversification arise from the interaction of viral intrinsic traits, host and vector evolutionary histories, agroecosystem structure, and human-mediated landscape transformation operating across multiple spatial and temporal scales (García-Arenal and McDonald, 2003; Jones, 2009). Mexico’s exceptional biocultural diversity and the coexistence of traditional, semi-industrial, and highly intensified agricultural systems generate a heterogeneous evolutionary landscape in which contrasting viral dynamics coexist and overlap.

**Figure 2:**
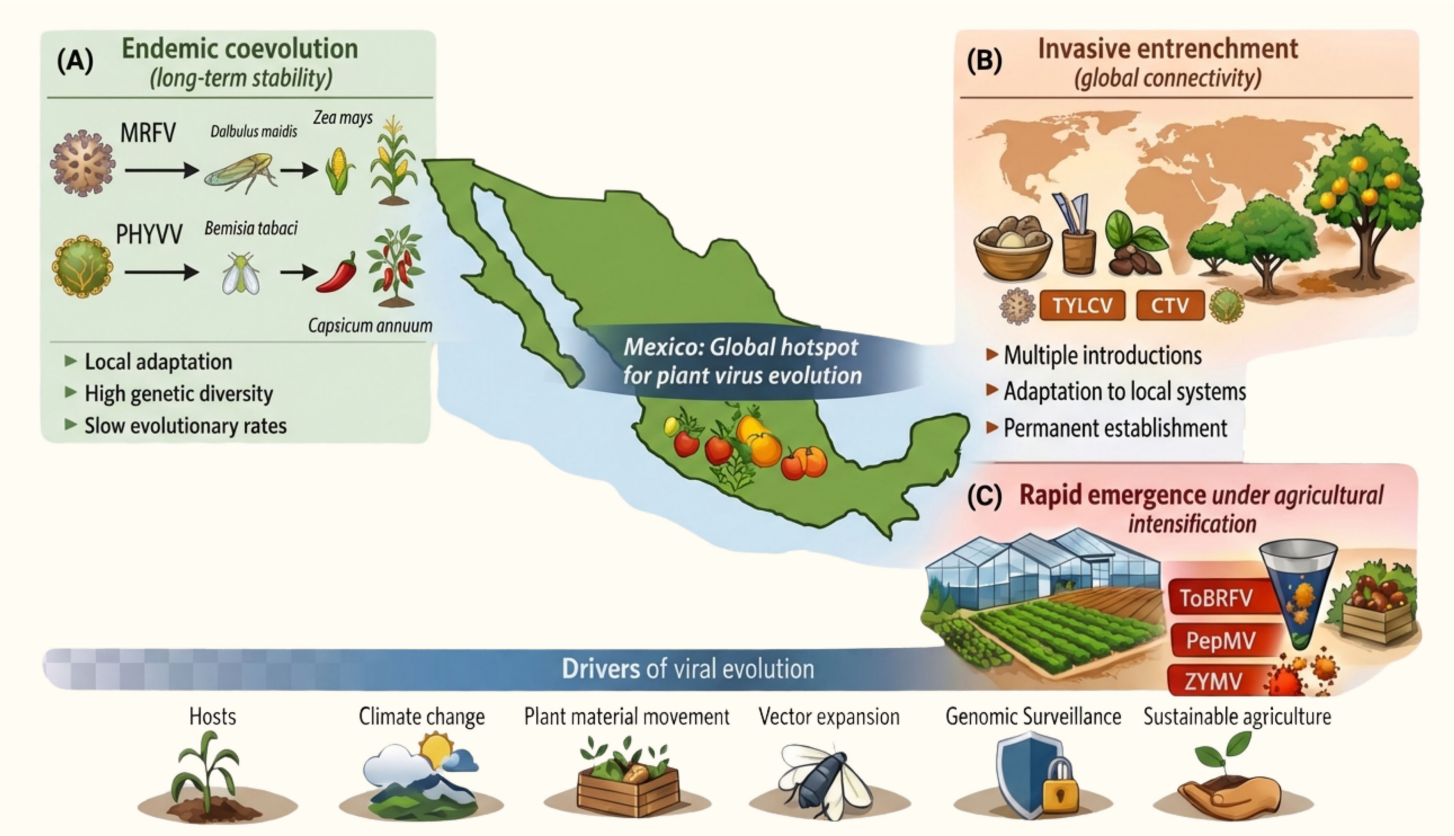
Eco-evolutionary regimes of plant virus emergence in Mexico. **(A)** Endemic coevolution. Long-term interactions among viruses, vectors, and hosts promote local adaptation and high genetic diversity, as observed for MRFV in maize (Dalbulus maidis) and PHYVV in pepper (Bemisia tabaci). **(B)** Invasive entrenchment. Globally introduced viruses, such as TYLCV and CTV, become established in new agroecosystems through repeated introductions, adaptation to local hosts, and efficient vector transmission. Created at https://BioRender.com.

We define eco-evolutionary regimes as recurring contexts characterized by specific constellations of selective pressures, transmission structures, and evolutionary timescales that jointly shape dominant patterns of viral diversification and epidemiological behavior (**Figure 2**, **Supplementary Table 1**). These regimes are not discrete categories but positions along a continuum that can overlap, interact, and transition as ecological conditions and agricultural practices change. Importantly, multiple regimes frequently intersect within shared landscapes, facilitating mixed infections, recombination, and the emergence of locally adapted variants that cannot be inferred from single-virus perspectives.

The first regime corresponds to long-term endemic coevolution within Mesoamerican agroecosystems. Viruses such as MRFV and PHYVV exemplify this regime, having evolved in close association with anciently domesticated hosts and locally adapted vectors (Chicas et al., 2007; Rodelo-Urrego and García-Arenal, 2013). Evolutionary change proceeds gradually, structured by spatial heterogeneity, host diversity, and stable transmission networks, with diversification driven primarily by point mutations and fine-scale local adaptation rather than extensive recombination or long-distance dispersal. Epidemiologically, these viruses tend to remain regionally contained, producing persistent but non-explosive disease dynamics characteristic of diversified traditional agroecosystems.

A second regime reflects invasive introduction followed by evolutionary entrenchment. Viruses such as TYLCV and CTV entered Mexican agroecosystems via globalization-driven pathways, including international trade in propagative material and the expansion of competent vectors (Idris et al., 2007; Rivas-Valencia et al., 2020). Initial invasion phases were marked by rapid spatial spread and severe epidemics, followed by stabilization within perennial or continuously cultivated systems. Evolutionary dynamics under this regime are shaped by quasispecies behavior, recurrent transmission bottlenecks, and sustained selection imposed by host resistance, rootstock–scion combinations, and management practices (Folimonova and Sun, 2022). Over decadal timescales, these viruses transition from acute invaders to chronic agroecosystem components while continuing to generate virulent strains and resistance-breaking variants (Ramírez-Pool et al., 2022).

The third regime corresponds to rapid emergence under agricultural intensification, particularly in short-cycle, high-density cropping systems. Viruses such as ToBRFV and ZYMV typify this regime, where host genetic uniformity, continuous planting, intensive mechanical handling, and highly efficient transmission impose extreme selective pressures (Simmons et al., 2008). Molecular evolution is accelerated, with potyviruses exhibiting substitution rates on the order of ~10^-^³–10^-4^ substitutions per site per year, and tobamoviruses such as ToBRFV showing measurable change (~10^-5^–10^-6^ substitutions per site per year) sufficient for resistance breaking and host adaptation (Dombrovsky and Smith, 2017; Moury and Desbiez, 2020; Gomaa and Garcia-Ruiz, 2025). Frequent demographic bottlenecks associated with seed and mechanical transmission are counterbalanced by large effective population sizes during local amplification, collapsing evolutionary and epidemiological timescales and producing near-real-time viral evolution.

Situating these regimes within a broader global context highlights both shared mechanisms and regional distinctiveness. In the Southwest Australian Floristic Region (SWAFR), virus evolution is strongly structured by long-term biogeographic isolation, exceptional plant endemism, and agro–natural interfaces, where spillover between crops and native vegetation plays a dominant role and viral diversity is shaped by biodiversity filtering and conservation-driven dynamics rather than ancient domestication mosaics (Webster et al., 2007; Sadiq et al., 2024). In this system, native flora functions both as evolutionary reservoir and as recipient of crop-associated viral incursions, emphasizing cross-ecosystem transmission and ecological compartmentalization as primary drivers of diversification. In contrast to domestication-driven mosaics in Mexico, Australian viral diversity appears strongly structured by long-term biogeographic isolation and native biodiversity reservoirs (Sadiq et al., 2024) While in East Africa, by contrast, the emergence and diversification of cassava and sweetpotato virus complexes have been tightly linked to vegetative propagation, whitefly expansion, recombination, and continent-scale epidemic waves (Ndunguru et al., 2005; Tugume et al., 2023). In cassava mosaic disease systems, recombination among begomoviruses and high regional connectivity of planting material have generated rapid diversification and recurrent epidemic cycles (Ndunguru et al., 2005). Similarly, sweetpotato virus complexes in East Africa illustrate how synergistic coinfections and clonal propagation amplify resistance breakdown and accelerate adaptive dynamics (Tugume et al., 2023). In these agroecosystems, vegetative transmission networks and vector population dynamics function as primary evolutionary accelerators.

Beyond Australia and East Africa, Mexico also parallels other recognized evolutionary systems while remaining distinct in the spatial convergence of these dynamics. In the Andean region, virus diversification has been linked to centers of domestication and crop wild interfaces that sustain long-term host–virus associations under traditional agricultural mosaics (García-Arenal et al., 2001; Elena et al., 2014). In Mediterranean greenhouse systems, continuous cropping and global trade have accelerated the emergence of viruses such as Tomato brown rugose fruit virus, exemplifying intensification-driven evolution (Dombrovsky and Smith, 2017; Panno et al., 2020). Similarly, in East and Southeast Asia, the diversification of begomoviruses has been shaped by expanding Bemisia tabaci populations and high agricultural connectivity (De Barro et al., 2011; Lefeuvre et al., 2010).

Mexico differs not because these mechanisms are unique, but because they converge within a single bioculturally heterogeneous landscape. Centers of domestication, perennial systems, traditional milpa agriculture, and export-oriented intensification coexist and intersect, generating overlapping selective regimes that position Mexico as a composite evolutionary landscape rather than a singular model of virus emergence.

Crucially, these regimes should be interpreted as a *continuum* of evolutionary responses shaped by agroecological structure and human intervention rather than as isolated categories. Climate change, landscape homogenization, and increasing connectivity among production systems are likely to facilitate transitions between regimes, enabling endemic viruses to acquire invasive traits or entrenched viruses to re-emerge with altered virulence or host range (Farooq et al., 2022). While high-throughput sequencing has revealed complex viral population structures and widespread coinfections in Mexican agroecosystems (Pacheco-Dorantes et al., 2025; Zamora-Macorra et al., 2025a; Vásquez-Gutiérrez et al., 2024; Vásquez-Gutiérrez et al., 2025b), genomic detection alone does not equate to biological relevance. Situating viral populations within dominant eco-evolutionary regimes provides a predictive framework for interpreting genomic data, anticipating epidemic risk, and evaluating the durability of resistance strategies, not only in Mexico, but in comparable agroecological systems worldwide.

## Data Availability

The original contributions presented in the study are included in the article/**Supplementary Material**. Further inquiries can be directed to the corresponding author.
