# Supplementary material for: Mexico as a hotspot for plant virus evolution: eco-evolutionary regimes shaping viral emergence
Source: Front Plant Sci. 2026 Apr 7;17:1800439. doi: 10.3389/fpls.2026.1800439 (PMC13095680; doi:10.3389/fpls.2026.1800439)
Supplement: Supplementary file 1 [file Table1.docx]

**Tables**

**Supplemental Table 1. Eco-evolutionary signatures of representative plant viruses in Mexico**

| Virus | Evolutionary status | Genome type | Main vector(s) | Vector ecology | Dominant agroecosystem | Evidence of recombination | | Spatial scale |
| --- | --- | --- | --- | --- | --- | --- | --- | --- |
| Maize rayado fino virus (MRFV) | Endemic | ssRNA (–) | *Dalbulus maidis* | Specialist | Traditional / diversified (milpa) | No | Regional Mesoamerica | |
| Pepper huasteco yellow vein virus (PHYVV) | Endemic | ssDNA | *Bemisia tabaci* | Generalist | Traditional / semi-intensive | Yes | Regional (Mexico) | |
| Tomato yellow leaf curl virus (TYLCV) | Introduced / entrenched | ssDNA | *Bemisia tabaci* | Generalist | Intensive open-field | Yes | Global | |
| Citrus tristeza virus (CTV) | Introduced / entrenched | ssRNA (+) | Aphids (*Toxoptera citricida*) | Generalist | Perennial orchards | Limited | Global | |
| Tomato brown rugose fruit virus (ToBRFV) | Emerging | ssRNA (+) | Mechanical / seeds / pollinators | Non-vector | Protected agriculture | No | Global | |
| Zucchini yellow mosaic virus (ZYMV) | Emerging | ssRNA (+) | Aphids | Generalist | Intensive cucurbit systems | Rare | Global | |
| Cucurbit aphid-borne yellows virus (CAbYV) | Emerging | ssRNA (+) | Aphids | Generalist | Intensive cucurbit systems | Yes | Regional–global | |
| Watermelon mosaic virus (WMV) | Re-emerging | ssRNA (+) | Aphids / mechanical | Generalist | Intensive cucurbit systems | Yes | Global | |
| Lettuce big-vein associated virus (LBVaV) | Emerging | ssRNA (–) | *Olpidium brassicae*(fungal) | Specialist | Intensive horticulture | Limited | Regional | |
| Pepino mosaic virus (PepMV) | Emerging | ssRNA (+) | Mechanical / seed | Non-vector | Protected agriculture | No | Global | |
